# An uncommon presentation of Granulomatosis with Polyangiitis

**DOI:** 10.31138/mjr.29.1.49

**Published:** 2018-03-19

**Authors:** Eleftherios Pelechas, Georgios Zouzos, Paraskevi V. Voulgari, Alexandros A. Drosos

**Affiliations:** Rheumatology Clinic, Department of Internal Medicine, Medical School University of Ioannina, Ioannina, Greece

**Keywords:** Granulomatosis with Polyangiitis, subclinical coeliac disease, hepatosplenomegaly

## Abstract

In this case, we present a patient with an uncommon presentation of Granulomatosis with Polyangiitis (GPA) with hepatic involvement and the possible association with subclinical coeliac disease. We discuss the differential diagnosis and the relevant therapy.

## INTRODUCTION

Granulomatosis with Polyangiitis (GPA) is a systemic necrotizing vasculitis characterised by segmental inflammation and necrosis of blood vessels. It mainly affects the medium and small-calibre arteries, especially of the respiratory tract and kidneys.^1^ The clinical manifestations of the disease vary considerably, and at times, it is difficult to differentiate between the various types of necrotizing arteritis and in addition, it may present with an overlapping syndrome complicating even more the clinical picture.^2^ In this direction, we present a patient with an uncommon presentation involving liver and spleen pathology, but also positivity to coeliac disease serologic tests.

## CASE DESCRIPTION

We present a 35-year-old man with a rather non-significant past medical history apart from an episode of sialolithiasis in the past year and a recent upper airway infection. On his arrival in the emergency department he complained about fevers (mainly during nighttime – he was using paracetamol and non-steroidal anti-inflammatory drugs), diaphoresis, weight loss, generalised arthralgias and myalgias for almost one month. At admission, his temperature was 38,2°C, pulse was 83 beats/min, respiratory rate was 20 breaths/min, blood pressure was 131/77 mmHg and SpO2 95%. He was not in severe distress. The main findings from the clinical examination were decreased breath sounds of the lung bases, palpable but non-painful lymph nodes in the axillae and cervical regions as well as hepatosplenomegaly. The remainder of the examination was unremarkable. Laboratory studies showed elevated C-reactive protein 79 mg/l (0–6), erythrocyte sedimentation rate 42 mm/1^st^ hour (0–20), elevated aspartate aminotransferase and alanine aminotransferase 123 U/L and 77 U/L respectively (10–35), γ-glutamyl transferase 144 U/L (10–52), alkaline phosphatase 254 U/L (35–125) and aldolase 11,8 U/L (0–7,7). Full blood count and urea and electrolytes were within normal limits. A urine dip test was positive for protein and haemoglobin +2/+2 respectively. Two days after his admission to the hospital he developed abdominal pain, but he denied nausea.

**Figure 1.**
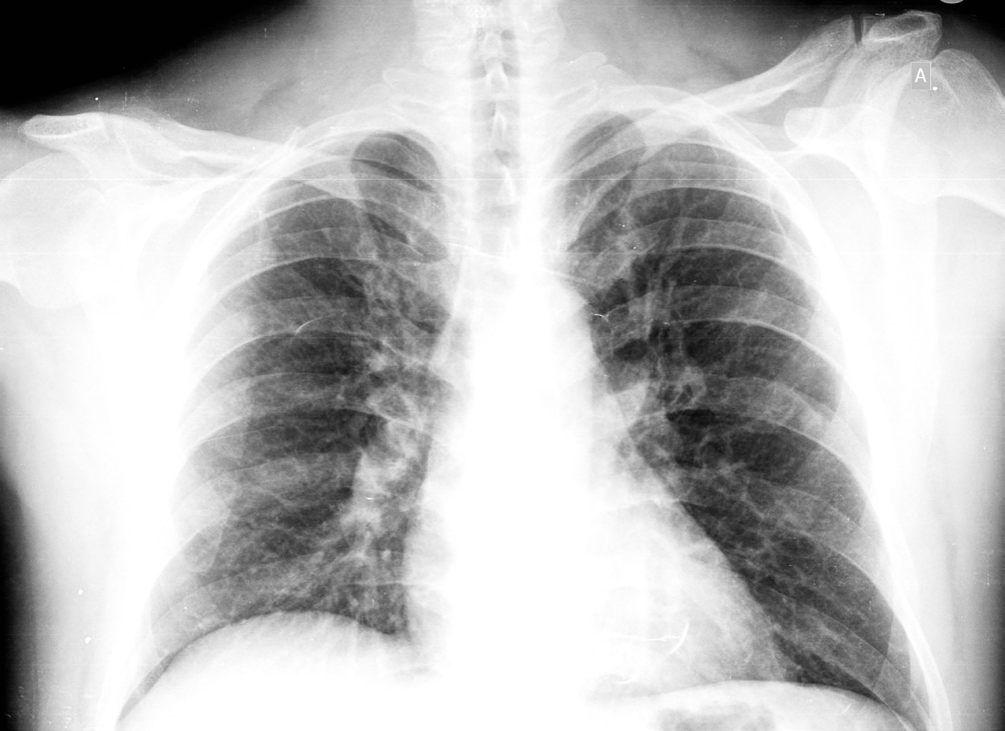
Chest x-ray on admission with no apparent findings.

**Figure 2.**
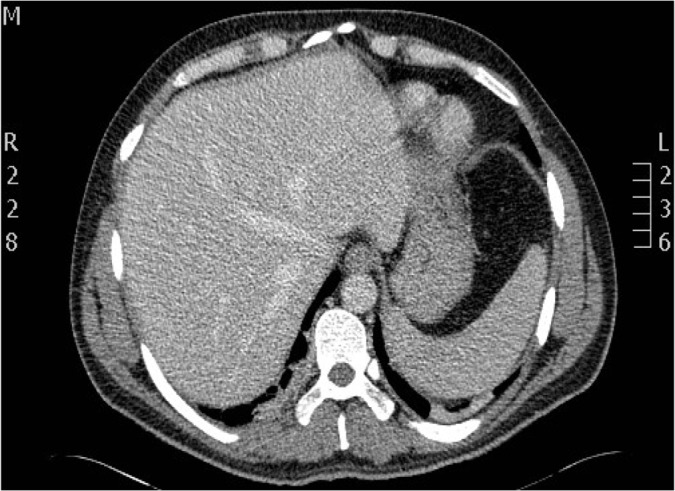
Computed Tomography of the abdomen confirming the hepato- and splenomegaly.

**Figure 3.**
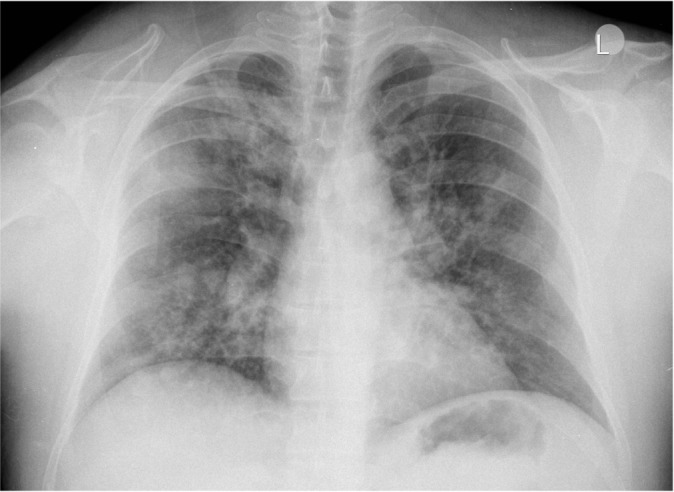
Multiple infiltrates on a chest x-ray

**Figure 4.**
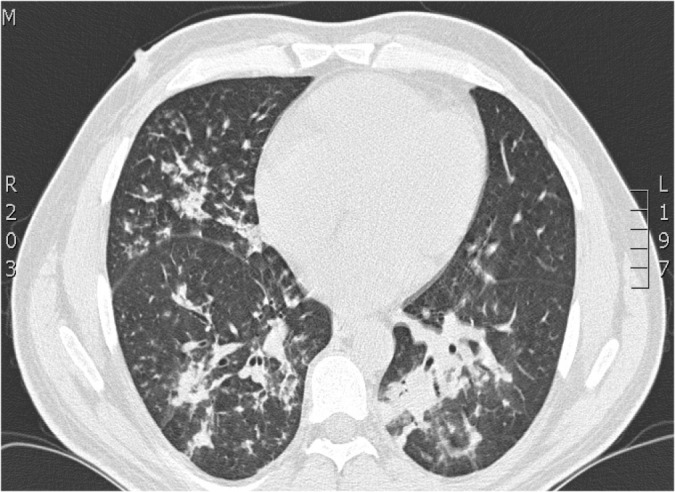
Computed Tomography of the lungs showing multiple nodules and ground glass areas

Based on the initial clinical and laboratory findings as well as the symptomatology, infective agents should be excluded. Blood and urine cultures, tuberculin skin test and interferon-γ release assay, B and C hepatitis, HIV I+II test were all negative. Also, serological tests for syphilis, leishmaniasis, brucellosis, Epstein-Barr and cytomegalovirus were also negative. Chest x-ray (CXR) on admission had no apparent findings (**Figure 1**). An abdominal ultrasonography and later a computed tomography (CT) of the abdomen confirmed the hepatosplenomegaly (**Figure 2**). Electromyography was normal. A liver biopsy showed non-specific inflammation with no fibrosis. Upper and lower gastrointestinal examination revealed also non-specific findings, but a small intestine biopsy came back with results compatible with coeliac disease. Further serologic tests for coeliac disease (anti-tissue transglutaminase, endomysial and deamidated gliadin peptide antibodies) were positive, thus, in lack of symptom-atology, a diagnosis of subclinical coeliac disease was made. A 24-hour urine collection was initially 1,6gr but reached up to 5gr/24h. Pulmonary function tests as well as diffusing capacity of the lung for carbon monoxide were within normal limits. Bronchoscopy was arranged but the patient developed dyspnoea and later haemoptysis. A new CXR revealed scattered lung infiltrates (**Figure 3**) and a lung CT showed multiple nodules and ground glass areas (**Figure 4**). At the same time, the immunologic evaluation came back with positive anti-neutrophil cytoplasmic antibodies (c-ANCA) at a titre of 1/80, proteinase-3 (PR-3) positive. Later, the renal biopsy revealed pauci-immune necrotic glomerulonephritis with crescent formation.

The patient was treated with 3 pulses of methylprednisolone (1gr/day) followed by prednisolone 1mg/kg/day and prophylaxis for osteoporosis. Also, pulses of cyclophosphamide were initiated at a dosage scheme of 0,5gr/m^2^/15 days for three months and steroid tapering with significant improvement.

## DISCUSSION

GPA or formerly known Wegener’s Granulomatosis was first described by Klinger^3^ in 1931, but in 1936, Wegener incorporated both clinical and histological criteria to describe what he believed represented a unique and distinctive syndrome.^4^ Virtually any organ can be involved but given the rarity of GPA, and non-specificity of symptoms, the diagnosis is often missed and a judgement can only be made on a step-by-step clinical basis. In older studies, hepatic involvement was not a feature of GPA, however, isolated case reports have been published mentioning of hepatic involvement^5–6^ in GPA patients.

Goritsas et al^6^ in a case report with a GPA patient favor the hypothesis that hepatic vasculitis may be the cause of acute hepatocellular necrosis, because there was immediate response of liver function tests after the initiation of immunosuppressive therapy with prednisone and cyclophosphamide. Carr et al^7^ has confirmed cytotoxic T lymphocyte-associated molecule-4 (CTLA4) and protein tyrosine phosphatase, non-receptor type-22 (PTPN22) as susceptibility loci in ANCA-associated vasculitides (AAV). These genes encode two key regulators of the immune response and are associated with many autoimmune diseases, including type-1 diabetes, autoimmune thyroid disease, coeliac disease, rheumatoid arthritis, and AAV. Thus, there may be a common pathogenetic mechanism that strengthens the diagnosis of AAV with subclinical coeliac disease in our patient.

Although the aetiology of GPA remains unclear and the course of the disease is not always as it appears in the textbooks, GPA remains a real challenge for the clinician both for the diagnosis and the treatment.

## CONFLICT OF INTEREST

The authors declare no conflict of interest.
